# Seasonal dynamics in mosquito abundance and temperature do not influence avian malaria prevalence in the Himalayan foothills

**DOI:** 10.1002/ece3.3319

**Published:** 2017-09-03

**Authors:** Farah Ishtiaq, Christopher G. R. Bowden, Yadvendradev V. Jhala

**Affiliations:** ^1^ Centre for Ecological Sciences Indian Institute of Science Bangalore India; ^2^ Royal Society for the Protection of Birds, The Lodge Sandy Bedfordshire UK; ^3^ Wildlife Institute of India Dehradun Uttarakhand India

**Keywords:** *Haemoproteus*, mosquito abundance, *Plasmodium*, prevalence, temperature, western Himalaya

## Abstract

We examined seasonal prevalence in avian haemosporidians (*Plasmodium* and *Haemoproteus*) in migrant and resident birds in western Himalaya, India. We investigated how infection with haemosporidians in avian hosts is associated with temporal changes in temperature and mosquito abundance along with host abundance and life‐history traits (body mass). Using molecular methods for parasite detection and sequencing partial cytochrome *b* gene, 12 *Plasmodium* and 27 *Haemoproteus* lineages were isolated. Our 1‐year study from December 2008 to December 2009 in tropical Himalayan foothills revealed a lack of seasonal variation in *Plasmodium* spp. prevalence in birds despite a strong correlation between mosquito abundance and temperature. The probability of infection with *Plasmodium* decreased with increase in temperature. Total parasite prevalence and specifically *Plasmodium* prevalence showed an increase with average avian body mass. In addition, total prevalence exhibited a U‐shaped relationship with avian host abundance. There was no difference in prevalence of *Plasmodium* spp. or *Haemoproteus* spp. across altitudes; parasite prevalence in high‐altitude locations was mainly driven by the seasonal migrants. One *Haemoproteus* lineage showed cross‐species infections between migrant and resident birds. This is the first molecular study in the tropical Himalayan bird community that emphasizes the importance of studying seasonal variation in parasite prevalence. Our study provides a basis for further evolutionary study on the epidemiology of avian malaria and spread of disease across Himalayan bird communities, which may not have been exposed to vectors and parasites throughout the year, with consequential implications to the risk of infection to naïve resident birds in high altitude.

## 1 INTRODUCTION

Parasite prevalence is a fundamental measure required to understand the temporal and spatial variations and epidemiology of infectious diseases (Ishtiaq, Rao, Huang, & Bensch, 2017; Scheuerlein & Ricklefs, 2004; Wood et al., 2007). The degree to which parasite prevalence varies depends on a suite of underlying mechanisms such as demographical (age, density, sex; Isaksson et al. 2013; Ricklefs et al., 2005), ecological (season, habitat quality, elevation; Rooyen, Lalubin, Glaziot, & Christe, 2013; Wood et al., 2007), and life‐history traits (body mass, fitness; Fecchio et al. 2011, Fecchio et al. 2013; González et al. 2014; Lutz et al. 2015; Matthews et al. 2016; Ricklefs et al., 2005; Svensson‐Coelho et al. 2013). The seasonal dynamics and geographic distributions of vectorborne parasites are strongly governed by seasonal changes in vector abundance (Chavasse et al., 1999; Emerson, Bailey, Mahdi, Walraven, & Lindsay, 2000; Lord, Woolhouse, Heesterbeek, & Mellor, 1996; Mabaso, Craig, Vounatsou, & Smith, 2005; Randolph, 2004; Sturrock et al., 2001). For example, seasonal variation in mosquito abundance in response to annual variation in temperature and rainfall can cause strong seasonal patterns of disease incidence in malaria‐epidemic regions, such as the Kenyan highlands (Hay et al. 2002). However, the role of temperature and vectors in explaining seasonal patterns in avian haemosporidian prevalence has been relatively under studied (see LaPointe et al. 2005; Medeiros et al. 2015; Okanga, Cumming, & Hockey, 2013).

Avian haemosporidians in the genera *Plasmodium* and *Haemoproteus* (Phylum Apicomplexa, Order Haemosporida) are globally distributed vector‐mediated parasites found in a broad range of birds (Valkinũas, 2005). *Plasmodium* and *Haemoproteus* parasites reproduce sexually in dipteran vectors—culicid mosquitoes and ceratopogonid midges, respectively (Valkinũas, 2005)—and use birds as intermediate hosts in which they undergo asexual reproduction. Parasites in both genera have been shown to be pathogenic to their avian hosts (Atkinson & van Riper, 1991; Bennett et al. 1993; Warner 1968) with deleterious effects on health and reproductive success (e.g., Marzal, De Lope, Navarro, & Møller, 2005) and body condition (Valkinũas, Zickus, Shapoval, & Iezhova, 2006), and possibly lead to extinction in immunologically naive hosts (Atkinson, Dusek, Woods, & Iko, 2000; Samuel et al., 2011). Thus, these parasites play a significant selective factor in bird populations and exert strong selection pressure on host life‐history traits.

The incubation period of malaria parasites within mosquitoes is exquisitely temperature sensitive (Paaijmans, Read, & Thomas, 2009); therefore, temperature is a major determinant of malaria transmission and persistence in host populations (LaPointe et al. 2005). Low ambient temperatures prevent sporogonic development of malaria parasites within vectors, influencing their altitudinal distributions (LaPointe, Goff, & Atkinson, 2010). Similarly, change in habitat composition from wet to dry due to climatic shift may alter both the geographic range and local abundance of malaria pathogens because their vectors (mosquitoes, Ceratopogonid midges, and Simuliid flies) require wet habitat to complete their life cycle (Valkinũas, 2005). Furthermore, owing to this vector dependency, both human (Hay et al., 2002) and avian (Cosgrove, Wood, & Sheldon, 2008) *Plasmodium* show a marked seasonality in transmission which appear to result in skewed prevalence estimates in winters. However, such stark variation in seasonal patterns ought to be more prominent in temperate regions where a peak in malaria prevalence occurs in late summer and autumn when the proportion of juveniles coincides with a rise in vector populations (see Beaudoin, Applegate, David, & McLean, 1971; Cranston, Ramsdale, Snow, & White, 1987; Marshall, 1938). In tropical and subtropical climates, malaria parasites transmission can occur year‐round (Valkinũas, 2005). In Hawaii, for example, malaria transmission occurs after the breeding season as chronically infected native birds serve as year‐round reservoir of disease (LaPointe, Atkinson, & Samuel, 2012). However, within tropical and subtropical areas, transmission will not occur at very high altitudes or during the cold season. There is a lack of quantitative studies which limits our capacity to understand and predict such changes in other threatened ecosystems and has largely remained unexplored in the tropics and in particular in the Indian subcontinent, despite it being the major staging and wintering ground of the Central Asian Flyway populations.

The Himalayan mountain range is one of the most species‐rich areas in the world, harboring about 8% of the world's bird species (Price et al. 2003). The high species diversity is due to species turnover associated with altitudinal variation in habitat, as well as variation in species composition along the range (Martens & Eck 1995; Price et al. 2003) and the possibility that such regions are buffered from climatic extremes, for example, because habitats and species can shift altitudinally in response to climate change (Fjeldså 1995; Fjeldså & Rahbek 2006). The Indian western Himalaya is species rich but remains a relatively understudied biogeographic region with only a handful of studies on birds (e.g., Price 1991), plants (Oommen and Shankar 2005), and mosquitoes (Devi and Jauhari 2004), with no previous studies on the dynamics of avian diseases and their vectors. Given the effect of climate and host demography which play a crucial role in determining parasite prevalence, as well as the dynamics of parasite transmission and host migration patterns both of which can increase the risk of infection. Many migrants move between altitudes or to the plains, and thereby encounter diverse parasite and vector faunas compared with the resident counterparts which remain at high altitudes throughout. Given that suitable vectors are present to transmit and maintain the infection, migrants can form an effective bridge for parasites between wintering and breeding grounds, hence increasing the risk of infection to naïve resident birds at the higher altitudes.

In this first molecular study on *Plasmodium* and *Haemoproteus* prevalence across resident and migrant birds, we aimed to explore the seasonal dynamics in parasite prevalence and its association with seasonal environmental drivers and host life‐history traits. Specifically, we examine (1) how mosquito abundance and temperature relate with *Plasmodium* spp. prevalence in foothills birds; (2) the effect of altitude on haemosporidian prevalence and diversity; and (3) the extent of cross‐species infections between resident and migrant birds.

## 2 METHODS

### 2.1 Study site and bird blood sampling

**Figure 1:**
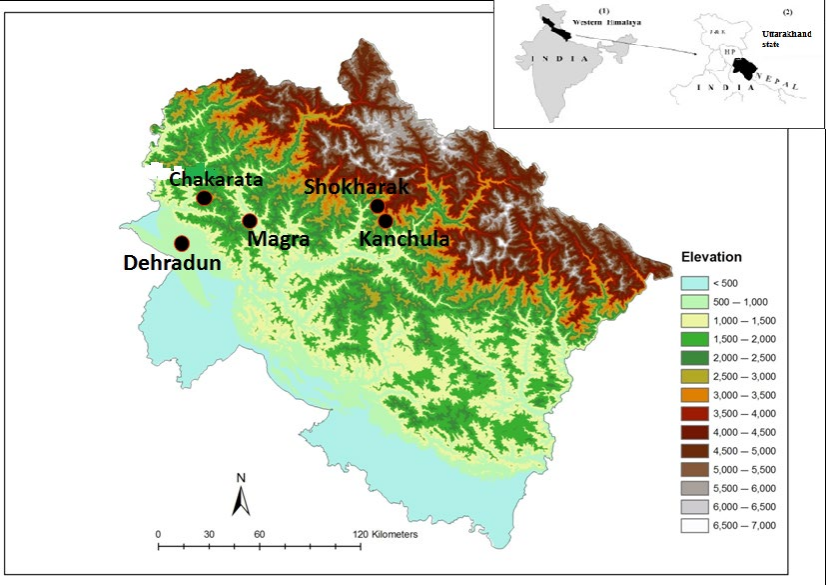
Bird sampling sites in Uttarakhand state, western Himalaya, India

From December 2008 to December 2009, we conducted bird blood sampling in Dehradun [DUN: 30.17409°N, 77.582535°E; 640 m above sea level (a.s.l.) inside the campus of Wildlife Institute of India, *n *=* *413]. The Wildlife Institute of India (WII) campus is nearly 200 acres of mixed habitat in the southern valley of Dehradun (Uttarakhand). To compare the patterns in parasite prevalence with high‐altitude breeding birds with foothills in April‐May 2009, in addition to DUN (*n *=* *38), we conducted sampling across three additional sites in forested habitat ranging between 1,800 and 3,200 m altitudes within Uttarakhand state, 2) Magra [MAG: N30.45587°E78.16158°; 1,800 m a.s.l.; *n *=* *24), 3) Chakrata [CHAK: N30.702°E77.869°; 2,200 m a.s.l.; *n *=* *12], and 4) Shokharakh (SHOK: N30.47860°E79.217980°; 3,100 m a.s.l.; *n *=* *45; Figure 1).

At each location, we set up 10–12 mist nets in high bird activity locations, often along forest edges, footpaths, or off‐road nature trails. Mist nets were 38‐mm gauge, 2.6 m tall, and 6, 9, or 12 m long. Birds were sampled between 0540 and 1230, and nets were checked every 5–10 min. Birds were identified to species according to Rasmussen and Anderton (2005) and ringed. Wing length was measured (maximum wing‐cord) with a steel rule (±1 mm) and body mass recorded with an electronic balance (±0.1 g). All birds were measured by F.I. Captured individuals were released at the site immediately after processing. We sampled 20–40 μl of bird blood from the sub‐brachial wing vein (never exceeding 1% of the individual's body weight). All samples were stored in SET Buffer (20–40 μl in 500 μl buffer 0.15 mol/L NaCl, 0.05 mol/L Tris, 0.001 mol/L EDTA, pH 8.0) at room temperature and subsequently transferred to −20°C.

### 2.2 Mosquito sampling and temperature data across seasons in foothills

Mosquito sampling was conducted during January–December 2009 four times in each month using standard Centers for Disease Control and Prevention (CDC) miniature black light (UV) traps without dry ice baits. For all sampling, the traps were suspended within the forest canopy, no more than 2 m from the ground on trees (DiMenna et al., 2006; Okanga et al., 2013) around avian blood sampling (mist‐netting) sites. Traps were operated overnight starting at 1,800 hr and picked up next morning at 0700 hr. Mosquitoes were collected from traps in the morning and stored at –20°C before identification. The species and sex of all mosquito samples were determined using a morphological mosquito‐identification key (Barraud, 1934; Christophers, 1933), and sorted by species and date followed by DNA barcoding technique (Kumar, Rajavel, Natarajan, & Jambulingam, 2007). As only female mosquitoes are responsible for parasite transmission, we provide estimates of abundance for female mosquitoes. Daily temperature records were obtained from the on‐site WII weather station in Dehradun.

### 2.3 Molecular methods

DNA extractions were performed using phenol chloroform extraction method (Sambrook, Fritsch, & Maniatis, 1987) or ammonium acetate protocol (Nicholls, Double, Rowell, & Magrath, 2000). We screened all bird samples for the combined presence of parasites of the genera *Plasmodium* and *Haemoproteus* using a polymerase chain reaction (PCR) protocol designed to amplify a 160‐bp fragment of mitochondrial ribosomal RNA gene (rRNA) of avian haemosporidians (213F/372R; Beadell & Fleischer, 2005) followed by a restriction enzyme‐based assay.

For samples that screened positive for the 16S rRNA gene fragment of the parasites, we amplified the cytochrome *b* (cyt‐*b*) gene fragments ranging from 533, 477, or 351 bp following Beadell et al. (2004), Hellgren, Waldenström, and Bensch (2004), and Ishtiaq et al. (2006), respectively. Each plate accompanied a parasite positive control and also a negative control to examine for any potential contamination. We screened all parasite‐negative samples for bird DNA (for cyt‐*b* gene) following Dumbacher, Pratt, and Fleischer (2003). The resulting PCR products were then sequenced in both directions. Sequences were assembled, aligned, and edited using SEQUENCHER version 5.2. We then identified sequences to genus using their closest sequence matches in GenBank or MalAvi database (Bensch, Hellgren, & Pérez‐Tris, 2009). Novel lineages were defined as lineages that differed by one or more nucleotides from any lineage deposited in GenBank prior to this study. All sequences are deposited in GenBank accession numbers: MF565807‐MF565836 (Table S1).

### 2.4 Phylogenetic analysis

To explore ecological and evolutionary relationships, a model‐based approach was used following phylogenetic reconstruction using the maximum‐likelihood analysis on 27 *Haemoproteus* sequences isolated from western Himalayan birds as well as 46 reference sequences downloaded from MalAvi database (Bensch et al., 2009). The *Plasmodium* phylogeny included 12 sequences isolated from western Himalayan birds and 27 reference sequences downloaded from MalAvi database (Bensch et al., 2009). *Leucocytozoon majoris* (GenBank accession number: AY393804) served as an outgroup in both phylogenies. The maximum‐likelihood tree was constructed using Bayesian phylogenetics as implemented in BEAST version 1.4.3 (Drummond & Rambaut, 2007) using the most appropriate substitution model (GTR + G) according to the Akaike Information Criterion implemented MEGA version 5.2 (Tamura et al., 2011). We present a maximum clade credibility tree using a relaxed molecular clock approach (Drummond & Rambaut, 2007). Rates of substitution were drawn from a lognormal distribution, and Yule prior was used for branching rates. We conducted two runs of 20 million generations, each with sampling conducted every 1,000 generations. *Tracer* (Rambaut & Drummond, 2003) was used to assess convergence, and whether two chains were mixing and whether the estimated sample size (ESS) for each parameter was sufficient (ESS > 200) to obtain robust parameter estimates. Four million generations were discarded as burn‐in from each run, leaving a posterior distribution of 32,000 trees.

### 2.5 Statistical analysis

We used contingency table analyses using G tests for the heterogeneity in parasite prevalence across host species and families followed by partitioned analyses (Sokal & Rohlf, 1995).

### 2.6 Individual‐level traits

We used generalized linear mixed models (GLMMs, function glmer in lme4; Bates, Maechler, Bolker, & Walker, 2015) to assess whether individual infection was influenced by individual‐level trait such as body condition, as fixed effects and bird taxonomy (species nested in genus and genus nested in family) as a random effect. Body condition (or size‐corrected body mass) is measured as the residuals of a species‐specific log (mass) by log (wing length) regression, which is often used as a proxy for overall condition (Schulte‐Hostedde, Zinner, Millar, & Hickling, 2005).

### 2.7 Species‐level traits

We also used GLMMs to assess whether infection prevalence in each host species was influenced by species‐specific traits, including avian abundance, mean species body mass (g), and migratory status as fixed effect and avian families with ≥5 individuals with bird taxonomy (species nested in genus and genus nested in family). We derived abundance estimates for each species by a single experienced observer (CB) recording the maximum number of birds seen on a given fixed circuit on dates within each week. We ensured that for any given week, at least one circuit of the nature trail within the WII campus was completed, but in some weeks, it may have been as many as 3–4 times. For any species known to be migratory or simply scarce, the exact date was noted, but for the remainder, simply the maximum figure seen during the week was noted. This may sound obvious, but by doing this, on a weekly (or even monthly) basis, it not only flags up when migrants are arriving, but in some cases, it identifies species that are not generally known to be migrants, but clearly do undergo at least localized movements. This presence/absence information can be a first step to detecting such localized seasonal movements. The averaged weekly maximum count for two visits per month was used as an index of abundance of each species. Abundance estimates (which are effectively an index for many of the more cryptic species) represent the predicted number of individuals of a given species detected in ~2.5 hr of surveying.

### 2.8 Seasonal effects on parasite prevalence, mosquito abundance, and temperature

Samples were ordered by the season collected (spring, summer, monsoon, autumn, and winter). We used GLMMs to assess whether individual infection was influenced by season, mosquito abundance, or average monthly temperature as fixed effects, and bird taxonomy (species nested in genus and genus nested in family) as a random effect. Mosquito abundance was the mean number of mosquitoes caught, and this was calculated to provide comparability with months where mosquitoes were trapped less than four nights in a month. Pearson's product–moment correlations (*r*) were run between mosquito abundance and temperature. To explore variation in parasite prevalence, we plotted correlation between prevalence in winter against prevalence in spring–summer, including the bird species for which at least four individuals were sampled in both seasons.

### 2.9 Altitudinal effects on parasite prevalence and diversity

We used GLMMs to assess whether individual infection was influenced by altitude and migratory status (resident, seasonal migrant, and long‐distance migrant) as fixed effects, and bird taxonomy (species nested in genus and genus nested in family) as a random effect.

All models were specified with a binomial error distribution and logit link function. The significance of fixed effects was evaluated with Wald's chi‐square tests (Bolker et al., 2009). Analyses were conducted in R v. 3.0.1 (R Development Core Team, 2010; R: A language and environment for statistical computing).

## 3 RESULTS

### 3.1 Year‐round sampling in Himalayan foothills (Dehradun: 600 m)

**Figure 2:**
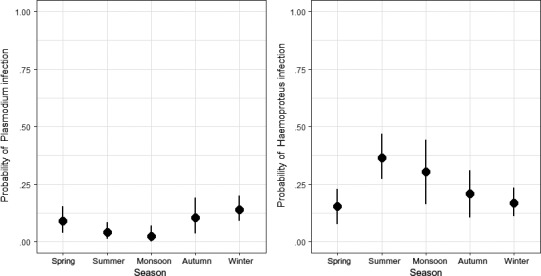
Comparison of *Plasmodium* and *Haemoproteus* prevalence across seasons in foothills (600 m; DUN) of western Himalaya

Among 413 bird blood samples representing 49 species from 19 families that were screened for infection with *Plasmodium* spp. or *Haemoproteus* spp., we detected an overall prevalence of 153 birds (37.68%) in which 24 species (49%) were infected. Of these, 37 were infected with *Plasmodium*, 95 were infected with *Haemoproteus,* and 21 were mixed infections. *Haemoproteus* spp. infections were more frequent than *Plasmodium* spp. infections (binomial test, *p* < .01; Figure 2). Overall haemosporidian prevalence was significantly heterogeneous among host species sampled with ten or more individuals (*G*adj = 115.92, *df* = 13, *p < *10^−5^) due to the high proportion of infected individuals in two species, *Zosterops palpebrosus* showed particularly high *Haemoproteus* prevalence (62.35%) followed by migrant *Acrocephalus dumetorum* (45%; Table 1). For host families sampled with five or more individuals, the total haemosporidian prevalence varied significantly (*G*adj = 115.92, *df* = 11, *p < *10^−5^).

**Table 1:** Seasonal prevalence and distribution of avian haemosporidian lineages across host family and species in the western Himalayan foothills, Dehradun

Scientific name	Host family	Spring		Summer		Monsoon		Autumn		Winter	
		No. infected	Lineage	No. infected	Lineage	No. infected	Lineage	No. infected	Lineage	No. infected	Lineage
*Prinia socialis*	Cisticolidae	2/5	P_DELURB5	0/6	0	0/1	0	0/2	0	2/7	P_GRW04; P_APR15
*Prinia hodgsoni*	Cisticolidae	0	0	0/1	0	0	0	0	0	2/4	P_UPUPA01
*Orthotomus sutorius*	Cisticolidae	1/3	P_UPUPA01	0/2				0/1		2/8	P
*Ploceus philippinus*	Ploecidae	0	0	0/2	0	0	0	0	0	1/3	H_PLOPHIL01
*Stachyris pyrrhops*	Timaliidae	2/7	P_GRW06	1/3	P	0/3	0	0/1	0	0/9	0
*Turdoides striata*	Timaliidae	0	0	1/2	H_TURSTR03	3/3	H_TURSTR02	0	0	6/6	H_TURSTR02; H_TURSTR04
*Pellorneum ruficeps*	Timaliidae	0/5	0	0/5	0	0/1	0	0	0	0	
*Pomatorhynius erythrogenys*	Timaliidae	0	0	2/3	H_ZOSPAL01	1/2	P_POMERY01	0	0	1/1	P_POMERY01
*Pnoepygya albiventor*	Timaliidae	0	0	0	0	0	0	0	0	0/1	
*Chrysomme sinense*	Timaliidae	0/1	0	0	0	0	0	2/5	P_DELURB5; P_GRW04	1/3	P
*Acrocephalus dumetorum*	Acrocephalidae	7/16	H_ACDUM1; H_ACDUM2; H_ACDUM3; H_MW1	11/14	H_ACDUM1; H_ACDUM2; H_ACDUM3	0/2	0	2/6	H_ACDUM1; H_MW1	0	0
*Cettia flavolivacea*	Sylviidae	1/1	P	0	0	0	0	0/1	0	0/2	0
*Sylvia curucca*	Sylviidae	1/1	H_WTH177	0	0	0	0	0	0	0	0
*Luscinia s. svecica*	Sylviidae	0	0	0	0	0	0	0	0	0/1	0
*Cettia brunnifrons*	Sylviidae	0	0	0	0	0	0	0	0	0/2	0
*Locustella major*	Sylviidae	0/2	0	0	0	0	0	0	0	1/1	0
*Phylloscopus coronatus*	Phylloscopidae	0/1	0	0	0	0	0	0	0	0	0
*Seicercus burkii*	Phylloscopidae	0/3	0	0	0	0	0	0	0	0	0
*Phylloscopus trochiloides*	Phylloscopidae	0	0	0/1	0	0	0	0	0	0	0
*Phylloscopus xanthoschistos*	Phylloscopidae	0	0	0	0	0	0	0/1	0	0/3	0
*Phylloscopus humei*	Phylloscopidae	0/1	0	0	0	0	0	0/1	0	0/1	0
*Phylloscopus chloronotus*	Phylloscopidae	0	0	0	0	0	0	0/1	0	0	0
*Phylloscopus tristis*	Phylloscopidae	0/2	0	0	0	0	0	0	0	0/3	0
*Phylloscopus fulgiventer*	Phylloscopidae	0	0	0	0	0	0	0	0	0/1	0
*Phylloscopus griseolus*	Phylloscopidae	0/1	0	0	0	0	0	0	0	0	0
*Parus major*	Paridae	0/3	0	0/1	0	0	0	0/2	0	0/3	0
*Saxicola ferreus*	Muscicapidae	0	0	0	0	0	0	0	0	0/4	0
*Luscinia pectoralis*	Muscicapidae	0/2	0	0	0	0	0	0/4	0	0	0
*Ficedula strophiata*	Muscicapidae	0	0	0	0	0	0	0	0	0/2	0
*Ficedula parva*	Muscicapidae	0	0	0	0	0/1	0	0	0	0	0
*Ficedula tricolor*	Muscicapidae	0	0	0	0	0	0	0/1	0	0/2	0
*Saxicola torquatus*	Muscicapidae	0	0	0	0	0	0	0	0	0/1	0
*Rhipidua albicollis*	Muscicapidae	0/3	0	0/5	0	0	0	0	0	0/1	0
*Pycnonotus leucogenys*	Pycnonotidae	2/2	H	0/2	0	0/2	0	4/8	P_PYCAFF01	0/4	0
*Pycnonotus cafer*	Pycnonotidae	1/1	H_PYCAFF02	1/3	P_PYCAFF01	0/1	0	0	0	7/17	H_ZOSPAL01; P_PYCAFF01
*Pycnonotus jocosus*	Pycnonotidae	0	0	0/1	0	0	0	0	0	0	
*Lanius schach*	Laniidae	0	0	0	0	0	0	0	0	0/2	
*Copscychus saularis*	Turdidae	0	0	0	0	0/1	0	0	0	0	
*Turdus unicolor*	Turdidae	1/1	P_AFTRU5	0	0	0	0	0	0	0	
*Zoothera citrina*	Turdidae	0	0	0/1	0	0	0	0	0	0	
*Sturnus contra*	Sturnidae	0	0	0	0	0	0	0	0	2/2	H_AFR084
*Amandava amandava*	Estrildidae	0/9	0	0/2	0	0/1	0	0/7	0	0/8	
*Lonchura punctulata*	Estrildidae	0/1	0	3/13	H_LONPUN01; H_LONPUN02	2/9	H_LONPUN01	0/2	0	4/7	H_LONPUN01; P_PYCAFF01
*Zosterops palpebrosus*	Zosteropidae	7/8	H_ZOSPAL01; H_ZOSPAL03; H_ZOSPAL04	21/29	H_ZOSPAL01; H_ZOSPAL02; H_ZOSPAL05	12/14	H_ZOSPAL01; H_ZOSPAL02	11/15	H_ZOSPAL01; H_ZOSPAL02	13/19	H_ZOSPAL01
H_ZOSPAL02											
P_DELURB5											

### 3.2 Influence of individual‐ and species‐level traits on parasite prevalence

**Figure 3:**
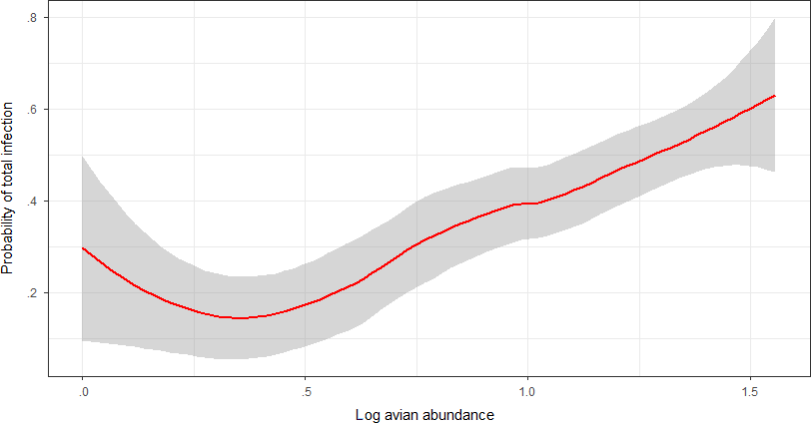
Relationship between total haemosporidian prevalence and avian abundance (log) in the foothills of western Himalaya. The regression includes species with sample sizes of more than four individuals

For individual‐level trait, infection status did not vary with body condition for *Plasmodium* spp. (Wald's χ² = 1.14, degrees of freedom (*df*) = 1, *p* = .28) nor for *Haemoproteus* spp. (Wald's χ² = 0.58, *df* = 1, *p* = .46). For species‐level traits, the log‐transformed avian abundance did not show significant effect on prevalence of *Plasmodium* spp. (Wald's χ² = 2.80, *df* = 1, *p* = .09; Table 2) and *Haemoproteus* spp. (Wald's χ² = 0.37, *df* = 1, *p* = .37; Table 2). However, total haemosporidian prevalence was marginally significant, increasing with host abundance (Wald's χ² = 4.17, *df* = 1, *p* < .04; Table 2; Figure 3).

**Table 2:** GLMM (generalized linear mixed model with binomial error distribution and logit link function) to test the influence of individual and species‐level traits along with environmental variables as fixed effects on *Plasmodium* and *Haemoproteus* infection rates after controlling for host taxonomy as random effect in birds sampled across seasons (*n* = 412) and altitudes (*n* = 119) in western Himalaya. Significant variables are in bold

	β	*SE*	*z* value	*p*
**A) Individual‐level trait**				
***Body Condition Index***				
Total infection	4.09	2.69	1.51	.12
*Plasmodium*	0.73	3.06	0.24	.80
*Haemoproteus*	2.52	3.53	0.71	.47
**B) Species‐level traits**				
***Avian abundance (log)***				
Total infection	1.05	0.51	2.04	**.04** a
*Plasmodium*	1.10	0.68	1.61	.10
*Haemoproteus*	0.72	0.51	1.40	.15
***Mean body weight (log)***				
Total infection	3.76	1.18	3.17	**.001** a
*Plasmodium*	0.81	0.34	2.34	**.01** a
*Haemoproteus*	1.95	1.18	1.62	.10
***Status (migrant versus resident)***				
Total infection	0.61	0.79	0.77	.44
*Plasmodium*	1.78	1.09	1.63	.10
*Haemoproteus*	0.50	1.59	0.31	.74
**C) ** ***Temperature***				
*Plasmodium*	−0.09	0.037	−2.55	**.01** a
*Haemoproteus*	0.03	0.027	1.09	.27
***D) Mosquito abundance (log)***				
*Plasmodium*	−0.67	0.33	−1.98	**.04** a
***E) Season***				
Total infection				
Spring (Intercept)	−1.21	0.51	−2.36	.01
Summer	−0.08	0.39	−0.21	.82
Monsoon	−0.36	0.50	−0.72	.47
Autumn	−0.39	0.45	−0.88	.37
Winter	−0.15	0.39	−0.39	.69
*Plasmodium*				
Spring (Intercept)	−2.79	0.62	−4.46	.01
Summer	−0.84	0.71	−1.17	.24
Monsoon	−1.62	1.16	−1.39	.16
Autumn	0.40	0.65	0.61	.53
Winter	0.17	0.54	0.32	.74
*Haemoproteus*				
Spring (Intercept)	−5.15	2.19	−2.35	.01
Summer	0.60	0.49	1.21	.22
Monsoon	0.10	0.59	0.18	.85
Autumn	−0.13	0.56	−0.24	.80
Winter	0.27	0.55	0.50	.61
***F) Altitude*** + ***status***				
Total infection^a^				
Altitude_600 m (Intercept)	3.28	1.82	1.79	.07
Status_resident	−4.47	1.82	−2.45	**.01**
Status_seasonal migrant	−2.49	1.48	−1.6	.09
Altitude_1,800 m	−1.73	1.14	−1.51	.12
Altitude_2,200 m	−0.75	1.29	−0.58	.56
Altitude_3,000 m	−3.21	1.60	−1.99	**.04**

a *Plasmodium* and *Haemoproteus* did not show significant results.

**Figure 4:**
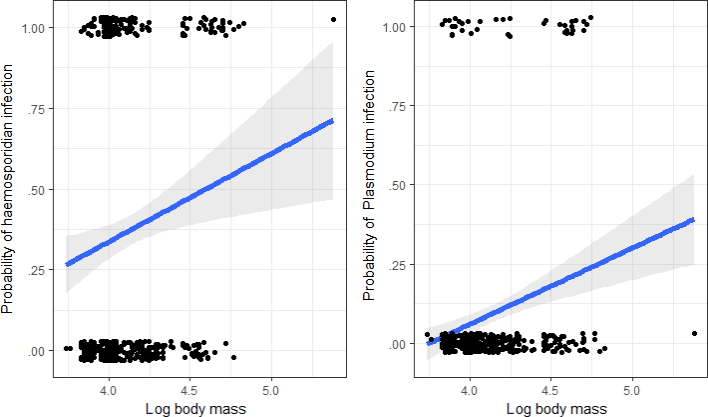
Relationship between haemosporidian prevalence and average body mass (log) in the foothills of western Himalaya

Both total haemosporidian prevalence (Wald's χ² = 7.44, *df* = 1, *p* < .01) and *Plasmodium* spp. prevalence (Wald's χ² = 4.96, *df* = 1, *p* < .02; Figure 4) increased with average species body mass, but *Haemoproteus* spp. (Wald's χ² = 2.65, *df* = 1, *p* = .10) prevalence showed no effect with body mass. There was no significant difference in prevalence of *Plasmodium* spp. (Wald's χ² = 3.49, *df* = 1, *p* = .75), *Haemoproteus* spp. (Wald's χ² = 0.17, *df* = 1, *p* = .89), or total prevalence (Wald's χ² = 2.29, *df* = 1, *p* = .12) between migrant and resident birds.

### 3.3 Seasonal effects on parasite prevalence and mosquito abundance

The total haemosporidian prevalence (Wald's χ² = 1.20, *df* = 4, *p* = .87), *Plasmodium* spp. prevalence (Wald's χ² = 7.07, *df* = 4, *p* = .13), and *Haemoproteus* spp. prevalence (Wald's χ² = 3.56, *df* = 4, *p* < .46; Figure 2) did not vary by season.

**Figure 5:**
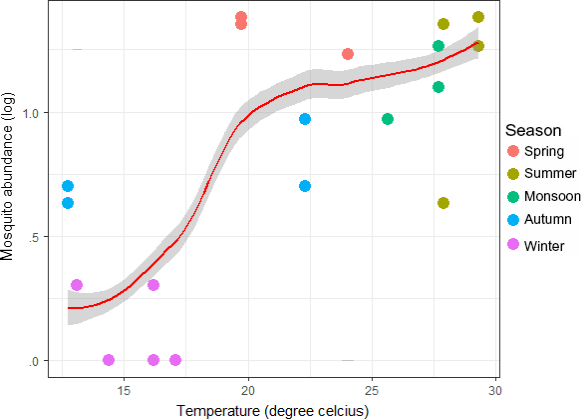
Relationship between mosquito abundance (log) with temperature (°C)

**Figure 6:**
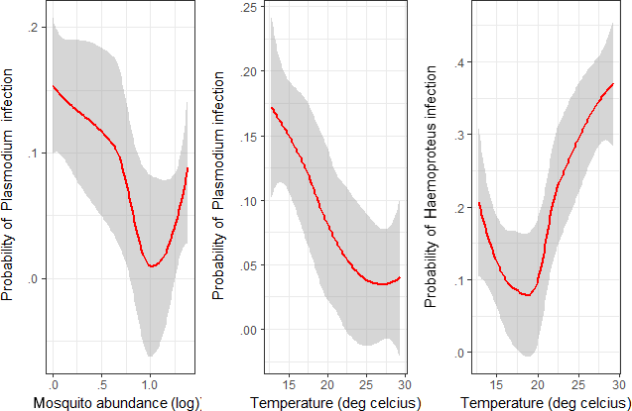
Relationship between temperature (°C) and probability of haemosporidian infection

Fifteen species representing six genera were identified from 588 specimens of mosquitoes. The *Culex* genus comprising of five species was the dominant group with *Culex quinquefasciatus* as the prevalent species which is a known vector of avian *Plasmodium*. There was significant correlation between the log mosquito abundance and temperature (*p* < .02; Figure 5), with change in mosquito species composition (Fig. S1). The abundance of *Culex* mosquito species dominated summer period and *Uranotenia* species in winter. The prevalence of *Plasmodium* spp. decreased (Wald's χ² = 7.30, *df* = 1, *p* < .01) with increase in temperature and mosquito abundance (Wald's χ² = 3.94, *df* = 1, *p* < .04; Table 2; Figure 6). Prevalence of *Haemoproteus* spp., however, showed no significant variation with temperature (Wald's χ² = 1.21, *df* = 1, *p* = .27). Temporal variation across six bird species suggested time‐independent species‐specific prevalence levels (*r *=* *0.76, *p *<* *.01; Table 1).

### 3.4 Seasonal sampling across altitudinal gradient

Of 119 bird samples representing 50 species from 17 families that were screened for *Plasmodium* spp. and *Haemoproteus* spp. infections, an additive model of altitude + status showed significant variation (Wald's χ² = 9.87, *df* = 2, *p* < .01). Total prevalence did not show variation across altitude (Wald's χ² = 5.35, *df* = 3, *p* = .14; Table 3). However, resident birds were marginally less infected (Wald's χ² = 6.01, *df* = 2, *p* < .49; Table 2) than seasonal migrants. There was no relationship detected between prevalence of *Plasmodium* spp. and *Haemoproteus* spp. when accounting for host taxonomy.

**Table 3:** Avian *Plasmodium* and *Haemoproteus* prevalence and distribution of lineages in bird species sampled in April–May across an altitudinal gradient

Scientific name	Host family	Status	DUN (600 m)		MAG (1,800 m)		CHAK (2,200 m)		SHOK (3,200 m)	
			No. infected	Lineage	No. infected	Lineage	No. infected	Lineage	No. infected	Lineage
*Prinia socialis*	Cisticolidae	R	0/3	0						
*Prinia hodgsoni*	Cisticolidae	R	0/1	0						
*Orthotomus sutorius*	Cisticolidae	R	0/2	0						
*Stachyris pyrrhops*	Timaliidae	R	2/4	P_GRW06						
*Turdoides striata*	Timaliidae	R	1/2	H_TURSTR03						
*Pellorneum ruficeps*	Timaliidae	R	1/4	P						
*Garrulax lanceolatus*	Timaliidae	SM			1/1	H_GARLAN01				
*Garrulax albogularis*	Leiothrichidae	SM					1/1	P_NILSUN01		
*Garrulax striata*	Leiothrichidae	SM			0/1					
*Minla strigula*	Leiothrichidae	SM							0/2	
*Trochalapteron erythrocephalum*	Leiothrichidae	SM							1/1	H_TROERY01
*Trochalapteron lineatum*	Leiothrichidae	SM							3/5	H_TROLIN01; P_TROLIN02
*Trochalapteron variegatum*	Leiothrichidae	SM							0/1	
*Acrocephalus dumetorum*	Acrocephalidae	M	18/30	H_ACDUM1; H_ACDUM2; H_ACDUM3; H_MW1						
*Aegithalos concinnus*	Aegithalidae	R			0/6		2/2	H_AEGCON01		
*Procarduelis nipalensis*	Fringillidae	SM							0/5	
*Carpodacus rodochroa*	Fringillidae	R							1/6	H_ACDUM1
*Aethopygea nipalensis*	Nectariniidae	SM							0/1	
*Phylloscopus occipitalis*	Phylloscopidae	SM	0/1	0	0	0				
*Seicercus burkii*	Phylloscopidae	SM	0/1	0	0	0			0/1	
*Phylloscopus trochiloides*	Phylloscopidae	M		0	3/4	H_GW1; H_GW7; H_GW8				
*Phylloscopus xanthoschistos*	Phylloscopidae	SM			0/1		0/2			
*Phylloscopus humei*	Phylloscopidae	SM					1/1	P		
*Phylloscopus maculipennis*	Phylloscopidae	R							0/5	
*Phylloscopus chloronotus*	Phylloscopidae	SM							0/4	
*Motacilla cinerea*	Motacillidae	M			1/2	H_YWT7				
*Anthus hodgsonii*	Motacillidae								0/1	
*Parus major*	Paridae	R	0/2	0						
*Parus monticolus*	Paridae	R			0/2		0/2		0/1	
*Sylviparus modestus*	Paridae	SM							0/6	
*Myophonus caeruleus*	Muscicapidae	SM	0	0	1/1	H_MYOCA01				
*Rhipidua albicollis*	Muscicapidae	R	0/4	0		0				
*Niltava macgrigoriae*	Muscicapidae	SM			0/1					
*Enicurus maculatus*	Muscicapidae				0/1					
*Tarsiger cyanurus*	Muscicapidae	SM					0/1			
*Ficedula superciliaris*	Muscicapidae	SM					0/1		0/1	
*Ficedula strophiata*	Muscicapidae	SM							0/2	
*Chaimarrornis leucocephalus*	Muscicapidae	R							0/2	
*Pycnonotus leucogenys*	Pycnonotidae	R	1/2	H		0				
*Pycnonotus jocosus*	Pycnonotidae	R	0/1	0		0				
*Picus squamatus*	Picidae	R			0/1					
*Dicrurus leucopheus*	Dicrurus	SM							0/1	
*Turdus unicolor*	Turdidae	SM	1/1	P_AFTRU5	0	0				
*Zoothera citrina*	Turdidae	SM	0/1	0		0				
*Amandava amandava*	Estrildidae	R	0/2	0		0				
*Lonchura punctulata*	Estrildidae	R	0/2	0						
*Glaucidium brodiei*	Strigidae	R	0	0	0/1	0				
*Zosterops palpebrosus*	Zosteropidae	R	4/7	H_ZOSPAL01; H_ZOSPAL03; H_ZOSPAL04						
*Yuhina flavicollis*	Zosteropidae	R			0/2					

M, migrant; R, resident; SM, seasonal migrant.

### 3.5 Cross‐species infections between migrant and resident birds

**Figure 7:**
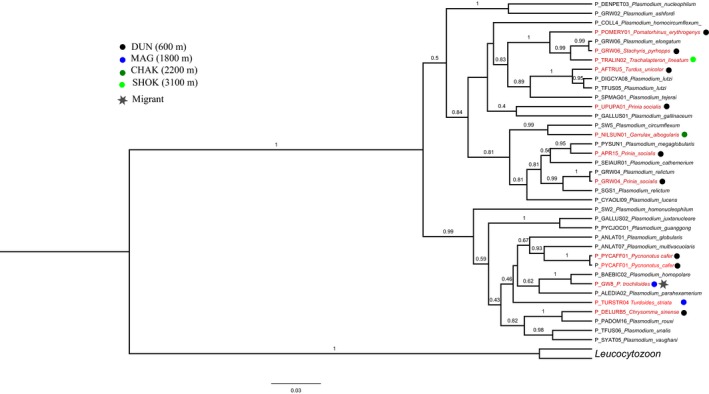
Maximum clade credibility tree of *Plasmodium* cyt‐*b* (477 bp) lineages recovered from western Himalayan birds in India. Posterior clade probability support values above 0.5 are shown. 12 *Plasmodium* lineages found in western Himalayan birds are in red text, and 27 reference *Plasmodium* lineages from MalAvi database (Bensch et al., 2009) are in black text

**Figure 8:**
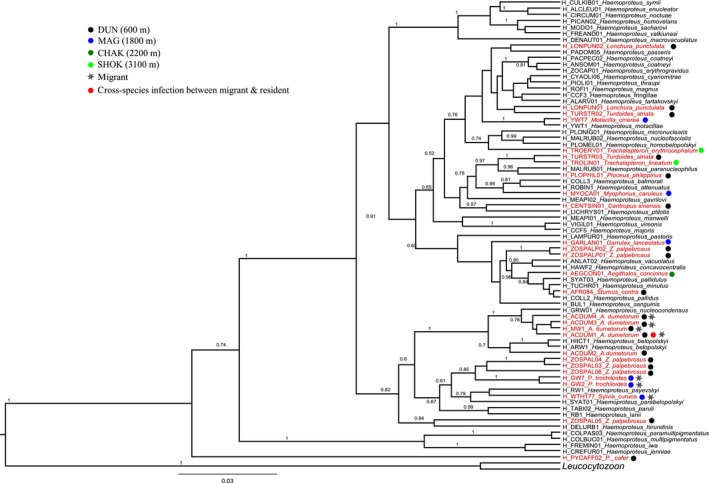
Maximum clade credibility tree of *Haemoproteus* cyt‐*b* (477 bp) lineages recovered from western Himalayan birds in India. Posterior clade probability support values above 0.5 are shown. 27 *Haemoproteus* lineages found in western Himalayan birds are in red text, and 46 reference *Haemoproteus* lineages from MalAvi database (Bensch et al., 2009) are in black text

Using PCR‐based detection methods, 37 cyt‐*b* sequences of parasite lineages were isolated, with a high proportion of infections of *Haemoproteus* spp.*,* representing 27 lineages whereas *Plasmodium* spp. had just 10 lineages (GenBank accession numbers are listed in Table S1). *Plasmodium* and *Haemoproteus* mitochondrial lineage relationships are presented independently in Figures 7 and 8, respectively. Most of the lineages found in the resident foothill birds were locally transmitted based on their presence in young birds hatched on‐site as well as the presence of lineages in the birds throughout the year. *Plasmodium* lineages found in the resident birds have been recorded earlier in host species with varying taxonomic affiliations whereas most of the *Haemoproteus* lineages recorded showed no overlap with parasite communities sampled elsewhere and shared within a host family. In particular, one *Haemoproteus* lineage (ACDUM1) sampled in Palearctic migrant warbler (*Acrocephalus dumetorum*) showed cross‐species infection with resident Himalayan species sampled at a high‐altitude site.

## 4 DISCUSSION

Our study is the first seasonal study in the tropics which captures the absence of seasonal pattern in prevalence of avian *Plasmodium* spp. These findings are in contrast with the Beaudoin et al. (1971) model for temperate areas that predicts a within‐year bimodal pattern of spring and autumn peaks with absence of infection in winter. Our 1‐year molecular study in tropical Himalayan foothills revealed a strong correlation between mosquito abundance and temperature; however, there was a lack of seasonal variation in *Plasmodium* spp. prevalence. There was no effect of body condition on parasite prevalence; however, log avian abundance contributed to variation in total haemosporidian prevalence. *Haemoproteus* spp. prevalence showed no variation across seasons and life‐history traits. Migrant birds were slightly more infected than resident species across altitudes.

### 4.1 Seasonal variation in parasite prevalence

Our year‐round study shows the lack of seasonal variation in parasite prevalence and its association with tropical climate which allows for the presence of mosquitoes and other hematophagous arthropod vectors and therefore facilitates avian malaria transmission throughout the year. Human malaria, however, shows a seasonal pattern in the Himalayan foothills with peak transmission from July–October (Dhiman, Chavan, Pant, & Pahwa, 2011). We did not find any peak in prevalence in spring due to a relapse of the previous year's infections (Beaudoin et al., 1971), which coincides with a peak in vector abundance (Atkinson & van Riper, 1991). In temperate regions, most *Haemoproteus* spp. show peak prevalence in the spring months when vectors (e.g., *Culicoides* spp.) become abundant or in the summer with acute infections in newly recruited immunologically naive juveniles (Cranston et al., 1987; Klei & DeGiusti, 1975; Marshall, 1938). It is very difficult to differentiate recrudescence and relapse in parasitemia in the tropics as the optimal conditions without any thermal constraints facilitate parasite transmission throughout the year. Furthermore, our use of sensitive PCR‐based screening methods provides a better estimate of parasite prevalence (Ishtiaq et al., 2017) and suggests that *Plasmodium* spp. and *Haemoproteus* spp. were indeed present in the blood throughout the year without undergoing a latent (dormant) phase where parasitemia survive in the tissues of avian hosts (Atkinson & van Riper, 1991). In temperate regions, dormancy is thought to minimize clearance from the host and to seasonally reduce virulence, thereby enhancing overwinter survival of the parasite (Worms, 1972). Re‐emergence of the parasite coincides with a peak in vector abundance. As our study is PCR‐based, we appreciate that parasite intensity data can shed more light on the dynamics of haemosporidian emergence with season providing insights into relapse of parasitemia by the presence of infective stages (gametocytes). The lack of temporal variation in parasite lineages across bird species suggests time‐independent species‐specific prevalence levels despite the change in temperature and mosquito species composition and abundance around the year.

### 4.2 Effect of host life‐history traits and environmental drivers on parasite prevalence

Prevalence of parasites varied significantly across host species and families. Of the three species‐level traits, we found no significant relationship between host body condition and infection status. However, both host abundance and mean body mass influenced the parasite prevalence in the foothills Himalayan bird community. These findings are similar to patterns in prevalence observed in temperate regions where species with the large body mass and at greater abundance are known to facilitate transmission and increase parasite prevalence (Matthews, Ellis, Roberts, Ricklefs, & Collins, 2015; Scheuerlein & Ricklefs, 2004). Large birds’ surface area provides more biting opportunities (Atkinson & van Riper, 1991) and emits more carbon dioxide which attracts host‐seeking vectors (Scheuerlein & Ricklefs, 2004). Epidemiological theory predicts that host density plays a central role in transmission of directly transmitted parasites (Anderson and May 1978). The positive relationship between host abundance and parasite prevalence is consistent with results from Dobson (2004) and Brown et al. (2001). In particular, the U‐shaped relationship between host abundance and parasite prevalence was similar to the temperate study by Ricklefs et al. (2005) where the most abundant host species had the highest infection prevalence followed by the least abundant host species. The high prevalence in our study was primarily driven by *Haemoproteus* spp. in highly abundant hosts: Zosteropidae, Acrocephalidae, Timaliidae, Cisticolidae families, whereas the least common host were migrant *Sylvid* warblers. High prevalence of *Haemoproteus* in hosts with high abundance can be explained by two underlying mechanisms: (1) high host‐to‐vector transmission rates in a densely populated community (Brown et al., 2001); (2) large numbers of susceptible hosts within a community will increase parasite transmission (Keesing et al. 2006). In our study, the high infection prevalence was a result of host‐specific *Haemoproteus* lineages which were shared within phylogenetically closely related species (see Ishtiaq, 2017). In contrast, Medeiros et al. (2015) found abundant hosts were more often bitten by mosquito vectors and were also more likely to be infected by *Plasmodium* parasites compared with less abundant hosts. In the Himalayan bird community, the vector–host encounter rates contribute to the positive relationships between host abundance and parasite prevalence; however, the temperature and mosquito abundance showed a negative effect on the *Plasmodium* spp. prevalence. The seasonal dynamics in parasite prevalence are a function of vector–host interactions leading to the transmission of parasites and vector abundance, host specificity, and ecological requirements of the vectors (van Riper et al. 1986). Temperature drives the transmission probability of parasites, which might be optimal in temperate regions only in summer, facilitating an increase in *Plasmodium* prevalence (e.g., Mederios et al. 2015). However, even in our tropical site, suboptimal conditions including the temperature threshold above which parasite cannot develop to infective stage within mosquito for a long period (e.g., 30°C for *P. relictum*; LaPointe et al., 2010), could result in a decrease in parasite prevalence. Temperature is an important determinant in malaria transmission, influencing both mosquito and parasite biology not only through variation in average temperature, but also by the extent of fluctuation in the daily temperature throughout the day (Blanford et al., 2013; Paaijmans et al., 2010) and noting that warmer temperatures reduces vectorial capacity of mosquitoes (Paaijmans et al., 2009). Given that incubation period of *Plasmodium* spp. within the mosquito is highly sensitive to a temperature threshold with development completely blocked under 15°C and over 30°C, thus potential of *Plasmodium* spp. transmission declines with increased temperature, despite a strong correlation between mosquito abundance and temperature.

### 4.3 Effect of altitude and seasonal migration on parasite prevalence

In contrast to studies in wet tropics and temperate regions (Atkinson et al., 2014; Rooyen et al., 2013; Zamora‐Vilchis, Williams, & Johnson, 2012) on altitudinal variation in haemosporidian distribution in bird community to single species model, we found no change in prevalence of *Plasmodium* spp. and *Haemoproteus* spp. across altitudes. The parasite prevalence in high‐altitude Himalayan sites is mainly driven by migrant hosts which move between altitudes or to the plains, and thereby encounter more diverse parasite and vector faunas compared with the resident counterparts occurring at high altitude which do not migrate to low altitudes. Avian haemosporidian parasites represent a complex, spatially heterogeneous host–parasite system, and their occurrence is strongly influenced and limited by environmental factors that affect vector prevalence and distribution. Seasonal movement of birds between altitudes or to the plains certainly adds to the complexity of the host–parasite associations, which warrants further investigation by longitudinal study across Himalayan sites.

### 4.4 Cross‐species infections

Many lineages observed in the *Haemoproteus* tree were host specific at a family level, which appears to be a trait in *Haemoproteus* species where the host ranges are often restricted to a limited number of closely related host species (Atkinson & van Riper, 1991; Bennett & Peirce, 1988; Perez‐Tris et al., 2007; Savage & Greiner, 2004). Among resident species sampled, *Zosterops palpebrosus* (Oriental white‐eye) was the most infected species with an assemblage of *Haemoproteus* lineages which appears to have diversified within the Zosteropidae family. Among migrants, *Acrocephalus dumetorum* was the most infected species with a diverse assemblage of *Haemoproteus belopolskyi*, which have a relatively low genetic distance from prevalent and widespread parasites of migrant Acrocephalids and Sylvids in Europe and African (Ishtiaq, 2017). This appears to be polymorphism within *H. belopolyskyi* clade. The *Haemoproteus* lineage ACDUM1 was shared with a pink‐browed rosefinch *Carpodacus rodochroa,* a high‐altitude species which points toward potential cross‐species transmission; however, as our study utilized only PCR, and not microscopy technique, there are several caveats associated with this methodology that could bias our lineage sharing results. First, the development of haemosporidians can be abortive in resistant or partly resistant hosts, resulting in no development of gametocytes (Cannell et al., 2013; Valkinũas, Ashford, Bensch, Killick‐Kendrick, & Perkins, 2011). Second, the low‐frequency infection could be spillover infections in resistant hosts leading to a dead end for parasite transmission, and indeed, this was confirmed with microscopy by the absence of gametocytes. Nonetheless, there was no sharing of parasite lineages between foothills and high‐altitude sites, which suggests demarcation of parasite transmission zones with a varying vector and thermal gradient. In *Plasmodium* tree, many lineages were generalists that infected multiple host species across multiple families.

Overall, this is the first molecular study of a tropical Himalayan bird community that reveals a high diversity of *Haemoproteus* lineages. While there was no seasonal variation in the prevalence of either *Haemoproteus* or *Plasmodium*, thus the lack of thermal constraints appears to facilitate year‐round transmission of these parasites. Many *Haemoproteus* lineages were host specific and have not been reported previously. These new data contribute to our understanding of seasonal dynamics of temperature, mosquito abundance, and thermal constraints in parasite transmission in the tropics. They also provide a basis for furthering our understanding on the ecology and epidemiology of avian malaria and the spread of disease across Himalayan bird communities, which may not generally be exposed to vector and parasites throughout the year. This can have important potential implications for the risk and susceptibility to infection for naïve resident birds at higher altitudes.

## ETHICAL APPROVAL

The field experiments comply with the current laws of the India where study was performed.

We thank Uttarakhand Forest Department for ethical approval and permission for collection of avian blood samples.

## CONFLICT OF INTEREST

The authors declare that they have no competing interests.

## AUTHOR CONTRIBUTION

FI conceived the idea. FI, CB, and YVJ designed the experiment; FI conducted the field and laboratory experiments; CB helped with fieldwork; FI wrote the manuscript, and FI, CB, and YVJ reviewed the manuscript. All authors approved the final version of the manuscript.

## References

[ece33319-bib-0503] Anderson, R. M. , & May, R. M. (1978). Regulation and stability of host‐parasite population interactions. I. Regulatory processes. Journal of Animal Ecology, 47, 219–247.

[ece33319-bib-0001] Atkinson, C. T. , Dusek, R. J. , Woods, K. L. , & Iko, W. M. (2000). Pathogenicity of avian malaria in experimentally infected Hawaii Amakihi. Journal of Wildlife Diseases, 36, 197–204. 10813599 10.7589/0090-3558-36.2.197

[ece33319-bib-0002] Atkinson, C. T. , Utzurrum, R. B. , LaPointe, D. A. , Camp, R. J. , Crampton, L. H. , Foster, J. T. , & Giambelluca, T. W. (2014). Changing climate and the altitudinal range of avian malaria in the Hawaiian Islands—An ongoing conservation crisis on the island of Kaua'i. Global Change Biology, 20, 2426–2436. 24446093 10.1111/gcb.12535

[ece33319-bib-0003] Atkinson, C. T. , & van Riper III, C. (1991). Pathogenicity and epizootiology of avian haematozoa: *Plasmodium*,* Leucocytozoon*, and *Haemoproteus* In Loye J. E., & Zuk M. (Eds.), Bird–parasite interactions: Ecology, evolution and behaviour (pp. 19–48). New York: Oxford University Press.

[ece33319-bib-0004] Barraud, P. J. (1934). The fauna of British India including Ceylon and Burma. Diptera vol. V. Family Culicidae Tribes Megarhinini and Culicini. London: Taylor & Francis.

[ece33319-bib-0005] Bates, D. , Maechler, M. , Bolker, B. , & Walker, S. (2015). Fitting linear mixed‐effects models using lme4. Journal of Statistical Software, 67, 1–48.

[ece33319-bib-0006] Beadell, J. S. , & Fleischer, R. C. (2005). A restriction enzyme‐based assay to distinguish between avian hemosporidians. Journal of Parasitology, 91, 683–685. 16108566 10.1645/GE-3412RN

[ece33319-bib-0007] Beadell, J. S. , Gering, E. , Austin, J. , Dumbacher, J. P. , Peirce, M. A. , Pratt, T. K. , … Fleischer, R. C. (2004). Prevalence and differential host‐specificity of two avian blood parasite genera in the Australo‐Papuan region. Molecular Ecology, 13, 3829–3844. 15548295 10.1111/j.1365-294X.2004.02363.x

[ece33319-bib-0008] Beaudoin, R. L. , Applegate, J. E. , David, D. E. , & McLean, R. G. (1971). A model for the ecology of avian malaria. Journal of Wildlife Diseases, 7, 5–13. 4400360 10.7589/0090-3558-7.1.5

[ece33319-bib-0500] Bennett, G. F. , Pierce, M. A. , & Ashford, R. W. (1993). Avian haematozoa: Mortality and pathogenicity. Journal of Natural History, 27, 993–1001.

[ece33319-bib-0009] Bennett, G. F. , & Peirce, M. A. (1988). Morphological form in the avian Haemoproteidae and an annotated checklist of the genus *Haemoproteus* Kruse, 1890. Journal of Natural History, 22, 1683–1686.

[ece33319-bib-0010] Bensch, S. , Hellgren, O. , & Pérez‐Tris, J. (2009). MalAvi: A public database of malaria parasites and related haemosporidians in avian hosts based on mitochondrial cytochrome *b* lineages. Molecular Ecology Resources, 9, 1353–1358. http://mbio-serv4.mbioekol.lu.se/avianmalaria/index.html 21564906 10.1111/j.1755-0998.2009.02692.x

[ece33319-bib-0011] Blanford, J. I. , Blanford, S. , Crane, R. G. , Mann, M. E. , Paaijmans, K. P. , Schreiber, K. V. , & Thomas, M. B. (2013). Implications of temperature variations for malaria parasite development across Africa. Scientific Reports, 3, 1300. 23419595 10.1038/srep01300 PMC3575117

[ece33319-bib-0012] Bolker, B. M. , Brooks, M. E. , Clark, C. J. , Geange, S. W. , Poulsen, J. R. , Stevens, M. H. , & White, J. S. (2009). Generalized linear mixed models: A practical guide for ecology and evolution. Trends in Ecology Evolution, 24, 127–135. 19185386 10.1016/j.tree.2008.10.008

[ece33319-bib-0013] Brown, C. R. , Komar, N. , Quick, S. B. , Sethi, R. A. , Panella, N. A. , Brown, M. B. , & Pfeffer, M. (2001). Arbovirus infection increases with group size. Proceedings of Royal Society London B Biological Sciences, 268, 1833–1840. 10.1098/rspb.2001.1749 PMC1088816 11522203

[ece33319-bib-0014] Cannell, B. L. , Krasnec, K. V. , Campbell, K. , Jones, H. I. , Miller, R. D. , & Stephens, N. (2013). The pathology and pathogenicity of a novel *Haemoproteus* spp. infection in wild little penguins (*Eudyptula minor*). Veterinary Parasitology, 197, 74–84. 23683654 10.1016/j.vetpar.2013.04.025

[ece33319-bib-0015] Chavasse, D. C. , Shler, R. P. , Murphy, O. A. , Huttly, S. R. A. , Cousens, S. N. , & Akhtar, T. (1999). Impact of fly control on childhood diarrhoea in Pakistan: Community‐randomised trial. Lancet, 353, 22–25. 10023946 10.1016/s0140-6736(98)03366-2

[ece33319-bib-0016] Christophers, S. R. (1933). The fauna of British India including Ceylon and Burma. Diptera vol. IV. Family Culicidae Tribe Anophelini. London: Taylor & Francis.

[ece33319-bib-0017] Cosgrove, C. L. , Wood, M. J. , & Sheldon, B. C. (2008). Seasonal variation in *Plasmodium* prevalence in a population of blue tits *Cyanistes caeruleus* . Journal of Animal Ecology, 77, 540–548. 18312339 10.1111/j.1365-2656.2008.01370.x

[ece33319-bib-0018] Cranston, P. S. , Ramsdale, C. D. , Snow, K. P. , & White, G. B. (1987). Adults, larvae and pupae of British mosquitoes (Culicidae). Ambleside: Freshwater Biological Association.

[ece33319-bib-0501] Devi, P. N. , & Jauhari, R. K. (2004). Altitudinal distribution of mosquitoes in mountainous areas of Garhwal region, Part I. Journal of Vector Borne Diseases, 41, 17–26. 15332482

[ece33319-bib-0019] Dhiman, R. C. , Chavan, L. , Pant, M. , & Pahwa, S. (2011). National and regional impacts of climate change on malaria by 2030. Current Science, 101, 372–383.

[ece33319-bib-0020] DiMenna, M. A. , Bueno, R. , Parmenter, R. R. , Norris, D. E. , Sheyka, J. , Molina, J. , … Glass, G. E. (2006). Comparison of mosquito trapping method efficacy for West Nile virus surveillance in New Mexico. Journal of American Mosquito Control Association, 22(2), 246–253. 10.2987/8756-971x(2006)22[246:comtme]2.0.co;2 PMC4152319 17019770

[ece33319-bib-0021] Dobson, A. (2004). Population dynamics of pathogens with multiple host species. The American Naturalist, 164, S64–S78. 10.1086/424681 15540143

[ece33319-bib-0022] Drummond, A. J. , & Rambaut, A. (2007). BEAST: Bayesian evolutionary analysis by sampling trees. BMC Evolutionary Biology, 7, 214. 17996036 10.1186/1471-2148-7-214 PMC2247476

[ece33319-bib-0023] Dumbacher, J. P. , Pratt, T. K. , & Fleischer, R. C. (2003). Phylogeny of the owlet‐nightjars (Aves: Aegothelidae) based on mitochondrial DNA sequence. Molecular Phylogenetics Evolution, 29, 540–549. 14615192 10.1016/s1055-7903(03)00135-0

[ece33319-bib-0024] Emerson, P. M. , Bailey, R. L. , Mahdi, O. S. , Walraven, G. E. L. , & Lindsay, S. W. (2000). Transmission ecology of the fly *Musca sorbens*, a putative vector of trachoma. Transactions of Royal Society of Tropical Medical Hygiene, 94, 28–32. 10.1016/s0035-9203(00)90427-9 10748893

[ece33319-bib-0504] Fecchio, A. , Lima, M. R. , Silveira, P. , Braga, E. M. , & Marini, M. A. (2011). High prevalence of blood parasites in social birds from a neotropical savanna in Brazil. Emu – Australian Ornithology, 111, 132–138.

[ece33319-bib-0505] Fecchio, A. , Lima, M. R. , Svensson‐Coelho, M. , Marini, M. A. , & Ricklefs, R. E. (2013). Structure and organization of an avian haemosporidian assemblage in a Neotropical savanna in Brazil. Parasitology, 1, 1–12. 10.1017/S0031182012001412 22939119

[ece33319-bib-0506] Fjeldså, J. (1995): Geographical patterns of neoendemic and older relict species of Andean birds: the significance of ecological stability areas In Churchill S. P., Balslev H., Forero E. & Luteyn J. L. (Eds.), Biodiversity and conservation of Neotropical montane forests (pp. 89–102). The New York Botanical Garden.

[ece33319-bib-0507] Fjeldsa, J. , & Rahbek, C. (2006). Diversification of tanagers, a species rich bird group, from lowlands to montane regions of South America. Integrative and Comparative Biology, 46, 72–81. 21672724 10.1093/icb/icj009

[ece33319-bib-0508] González, A. D. , Matta, N. E. , Ellis, V. A. , Miller, E. T. , Ricklefs, R. E. , & Gutiérrez, H. R. (2014). Mixed species flock, nest height, and elevation partially explain avian haemoparasite prevalence in Colombia. PLoS ONE, 9 (6), e100695 https://doi.org/10.1371/journal.pone 24950223 10.1371/journal.pone.0100695 PMC4065061

[ece33319-bib-0025] Hay, S. I. , Cox, J. , Rogers, D. J. , Randolph, S. E. , Stern, D. I. , Shanks, G. D. , … Snow, R. W. (2002). Climate change and the resurgence of malaria in the East African highlands. Nature, 415, 905–909. 11859368 10.1038/415905a PMC3164800

[ece33319-bib-0026] Hellgren, O. , Waldenström, J. , & Bensch, S. (2004). A new PCR assay for simultaneous studies of *Leucocytozoon*,* Plasmodium*, and *Haemoproteus* from avian blood. Journal of Parasitology, 90, 797–802. 15357072 10.1645/GE-184R1

[ece33319-bib-0600] Isaksson, C. , Sepil, I. , Baramidze, V. , & Sheldon, B. (2013). Explaining variance of avian malaria infection in the wild: The importance of host density, habitat, individual life‐history and oxidative stress. BMC Ecology, 13, 15. 23565726 10.1186/1472-6785-13-15 PMC3639228

[ece33319-bib-0027] Ishtiaq, F. (2017). Exploring host and geographical shifts in transmission areas of haemosporidians in a Palaearctic passerine wintering in India. Journal of Ornithology, https://doi.org/10.1007/s10336-017-1444-9 10.1007/s10336-017-1444-9 PMC6038909 30003008

[ece33319-bib-0028] Ishtiaq, F. , Beadell, J. S. , Baker, A. J. , Rahmani, A. R. , Jhala, Y. V. , & Fleischer, R. C. (2006). Prevalence and evolutionary genetics of haematozoan parasites in native versus introduced populations of common myna *Acridotheres tristis* . Proceedings of Royal Society London B Biological Sciences, 273, 587–594. 10.1098/rspb.2005.3313 PMC1560061 16537130

[ece33319-bib-0029] Ishtiaq, F. , Rao, M. , Huang, X. , & Bensch, S. (2017). Estimating prevalence of avian haemosporidians in natural populations—A comparative study on screening protocols. Parasites and Vectors, https://doi.org/10.1186/s13071-017-2066-z 10.1186/s13071-017-2066-z PMC5340044 28264710

[ece33319-bib-0601] Keesing, F. , Holt, R. D. , & Ostfeld, R. S. (2006). Effects of species diversity on disease risk. Ecology Letters, 9, 485–498. 16623733 10.1111/j.1461-0248.2006.00885.x

[ece33319-bib-0030] Klei, T. R. , & DeGiusti, D. L. (1975). Seasonal occurrence of *Haemoproteus columbae* Kruse and its vector *Pseudolynchia canariensis* Bequaert. Journal of Wildlife Diseases, 11, 130–135. 803576 10.7589/0090-3558-11.1.130

[ece33319-bib-0031] Kumar, P. N. , Rajavel, A. R. , Natarajan, R. , & Jambulingam, P. (2007). DNA barcodes can distinguish species of Indian Mosquitoes (Diptera: Culicidae). Journal of Medical Entomology, 44, 1–7. 17294914 10.1603/0022-2585(2007)44[1:dbcdso]2.0.co;2

[ece33319-bib-0602] LaPointe, D. A. , & M.L. Goff, C. T. Atkinson. (2005). Comparative susceptibility of introduced forest‐dwelling mosquitoes in Hawai'i to avian malaria, *Plasmodium relictum* . Journal of Parasitology, 91, 843–849. 17089752 10.1645/GE-3431.1

[ece33319-bib-0032] LaPointe, D. A. , Atkinson, C. T. , & Samuel, S. D. (2012). Ecology and conservation biology of avian malaria. Annals of the New York Academy of Sciences, 1249, 211–226. 22320256 10.1111/j.1749-6632.2011.06431.x

[ece33319-bib-0033] LaPointe, D. A. , Goff, M. L. , & Atkinson, C. T. (2010). Thermal constraints to the sporogonic development and altitudinal distribution of avian malaria *Plasmodium relictum* in Hawai'i. Journal of Parasitology, 96, 318–324. 20001096 10.1645/GE-2290.1

[ece33319-bib-0034] Lord, C. C. , Woolhouse, M. E. J. , Heesterbeek, J. A. P. , & Mellor, P. S. (1996). Vector‐borne diseases and the basic reproduction number: A case study of African horse sickness. Medical Veterinary Entomology, 10, 19–28. 8834738 10.1111/j.1365-2915.1996.tb00077.x

[ece33319-bib-0605] Lutz, H. L. , Hochachka, W. M. , Engel, J. I. , Bell, J. A. , Tkach, V. V. , Bates, J. M. , et al. (2015). Parasite prevalence corresponds to host life history in a diverse assemblage of afrotropical birds and haemosporidian parasites. PLoS ONE, 10(4), e0121254 https://doi.org/10.1371/journal.pone.0121254 25853491 10.1371/journal.pone.0121254 PMC4390322

[ece33319-bib-0035] Mabaso, M. L. H. , Craig, M. , Vounatsou, P. , & Smith, T. (2005). Towards empirical description of malaria seasonality in southern Africa: The example of Zimbabwe. Tropical Medical International Health, 10, 909–918. 10.1111/j.1365-3156.2005.01462.x 16135199

[ece33319-bib-0606] Martens, J. , & Eck, S. (1995). Towards an ornithology of the Himalayas: Systematics, ecology and vocalizations of Nepal birds. Bonner Zoologische Monographien, 38, 1–445.

[ece33319-bib-0036] Marshall, J. F. (1938). The British mosquitoes. London: British Museum.

[ece33319-bib-0037] Marzal, A. , De Lope, F. , Navarro, C. , & Møller, A. P. (2005). Malarial parasites decrease reproductive success: An experimental study in a passerine bird. Oecologia, 142, 541–545. 15688214 10.1007/s00442-004-1757-2

[ece33319-bib-0608] Matthews, A. E. , Ellis, V. A. , Hanson, A. A. , Roberts, J. R. , Ricklefs, R. E. , & Collins, M. D. (2016). Avian haemosporidian prevalence and its relationship to host life histories in eastern Tennessee. Journal of Ornithology, 157, 533–548.

[ece33319-bib-0609] Medeiros, M. C. , Ricklefs, R. E. , Brawn, J. D. , & Hamer, G. L. (2015). Plasmodium prevalence across avian host species is positively associated with exposure to mosquito vectors. Parasitology, 142(13), 1612–1620. 26394656 10.1017/S0031182015001183

[ece33319-bib-0039] Nicholls, J. A. , Double, M. C. , Rowell, D. M. , & Magrath, R. D. (2000). The evolution of cooperative and pair breeding in thornbills *Acanthiza* (Pardalotidae). Journal of Avian Biology, 31, 165–176.

[ece33319-bib-0701] Oommen, M. A. , & Shankar, K. (2005). Elevational species richness patterns emerge from multiple local mechanisms in Himalayan woody plants. Ecology, 85, 3039–3047.

[ece33319-bib-0040] Okanga, S. , Cumming, G. S. , & Hockey, P. A. R. (2013). Avian malaria prevalence and mosquito abundance in the western Cape, South Africa. Malaria Journal, 12, 370. 24160170 10.1186/1475-2875-12-370 PMC4016263

[ece33319-bib-0041] Paaijmans, K. P. , Blanford, S. , Bell, A. S. , Blanford, J. I. , Read, A. F. , & Thomas, M. B. (2010). Influence of climate on malaria transmission depends on daily temperature variation. Proceedings of Natural Academy of Sciences USA, 107, 15135–15139. 10.1073/pnas.1006422107 PMC2930540 20696913

[ece33319-bib-0042] Paaijmans, K. P. , Read, A. F. , & Thomas, M. B. (2009). Understanding the link between malaria and climate. Proceedings of Natural Academy of Sciences USA, 106, 13844–13849. 10.1073/pnas.0903423106 PMC2720408 19666598

[ece33319-bib-0043] Perez‐Tris, J. , Hellgren, O. , Krizanauskiene, A. , Waldenström, J. , Secondi, J. , Bonneaud, C. , … Bensch, S. (2007). Within‐host speciation of malaria parasites. PLoS One, 2, 1–7. 10.1371/journal.pone.0000235 PMC1794596 17311104

[ece33319-bib-0702] Price, T. (1991). Morphology and ecology of breeding warblers along an altitudinal gradient in Kashmir, India. Journal of Animal Ecology, 60, 643–664.

[ece33319-bib-0703] Price, T. , Zee, J. , Jamdar, K. , & Jamdar, N. (2003). Bird species diversity along the Himalayas: A comparison of Himachal Pradesh and Kashmir. Journal of the Bombay Natural History Society, 100, 394–409.

[ece33319-bib-0044] R Development Core Team (2010). R: A language and environment for statistical computing. Vienna, Austria: R Foundation for Statistical Computing.

[ece33319-bib-0045] Rambaut, A. , & Drummond, A. J. (2003). Tracer v1.3. Retrieved from http://evolve.zoo.ox.ac.uk/

[ece33319-bib-0046] Randolph, S. E. (2004). Tick ecology: Processes and patterns behind the epidemiological risk posed by ixodid ticks as vectors. Parasitology, 129, S37–S65. 15938504 10.1017/s0031182004004925

[ece33319-bib-0047] Rasmussen, P. C. , & Anderton, J. C. (2005). Birds of South Asia. The Ripley guide. Washington, DC and Barcelona: Smithsonian Institution and Lynx Edicions.

[ece33319-bib-0048] Ricklefs, R. E. , Swanson, B. L. , Fallon, S. M. , Martinez‐Abrain, A. , Scheuerlein, A. , Gray, J. , & Latta, S. (2005). Community relationships of avian malaria parasites in southern Missouri. Ecological Monograph, 75, 543–559.

[ece33319-bib-0049] Rooyen, J. , Lalubin, F. , Glaziot, O. , & Christe, P. (2013). Altitudinal variation in haemosporidian parasite distribution in great tit populations. Parasites and Vectors, 6, 139. 23648230 10.1186/1756-3305-6-139 PMC3658882

[ece33319-bib-0050] Sambrook, J. , Fritsch, E. F. , & Maniatis, T. (1987). Molecular cloning, a laboratory manual. Cold Spring Harbor: Cold Spring Harbor Laboratory Press.

[ece33319-bib-0051] Samuel, M. D. , Hobbelen, P. H. F. , DeCastro, F. , Ahumada, J. A. , LaPointe, D. A. , Atkinson, C. T. , … Duffy, D. C. (2011). The dynamics, transmission, and population impacts of avian malaria in native Hawaiian birds: A modeling approach. Ecological Applications, 21(8), 2960–2973.

[ece33319-bib-0052] Savage, A. F. , & Greiner, E. C. (2004). Hematozoa of the avian family Brachypteraciidae (the groundrollers). Journal of Parasitology, 90, 1468–1472. 15715245 10.1645/GE-227R

[ece33319-bib-0053] Scheuerlein, A. , & Ricklefs, R. E. (2004). Prevalence of blood parasites in European passeriform birds. Proceedings of Royal Society London B Biological Sciences, 271, 1363–1370. 10.1098/rspb.2004.2726 PMC1691737 15306334

[ece33319-bib-0054] Schulte‐Hostedde, A. I. , Zinner, B. , Millar, J. S. , & Hickling, G. J. (2005). Restitution of mass‐size residuals: Validating body condition indices. Ecology, 86, 155–163.

[ece33319-bib-0055] Sokal, R. R. , & Rohlf, F. J. (1995). Biometry: The principles and practice of statistics in biological research, 3rd ed. New York, W.H: Freeman.

[ece33319-bib-0056] Sturrock, R. F. , Diaw, O. T. , Talla, I. , Niang, M. , Piau, J. P. , & Capron, A. (2001). Seasonality in the transmission of schistosomiasis and in populations of its snail intermediate hosts in and around a sugar irrigation scheme at Richard Toll, Senegal. Parasitology, 123, S77–S89. 11769294 10.1017/s0031182001008125

[ece33319-bib-0604] Svensson‐Coelho, M. , Blake, J. G. , Loiselle, B. A. , Penrose, A. S. , Parker, P. G. , & Ricklefs, R. E. (2013). Diversity, prevalence, and host specificity of avian Plasmodium and Haemoproteus in a Western Amazon assemblage. Ornithology Monographs, 76, 1–47.

[ece33319-bib-0057] Tamura, K. , Peterson, D. , Peterson, N. , Stecher, G. , Nei, M. , & Kumar, S. (2011). MEGA 5: Molecular evolutionary genetics analysis using maximum likelihood, evolutionary distance, and maximum parsimony methods. Molecular Biology Evolution, 28, 2731–2739. 21546353 10.1093/molbev/msr121 PMC3203626

[ece33319-bib-0607] van Riper, C. , van Riper III, S. G. , Goff, M. L. , & Laird, M. (1986). The epizootiology and ecological significance of malaria in Hawaiian (USA) land birds. Ecological Monographs, 56, 327–344.

[ece33319-bib-0058] Valkinũas, G. , Ashford, R. W. , Bensch, S. , Killick‐Kendrick, R. , & Perkins, S. (2011). A cautionary note concerning *Plasmodium* in apes. Trends in Parasitology, 27(6), 231–232. 21497136 10.1016/j.pt.2011.02.008

[ece33319-bib-0059] Valkinũas, G. (2005). Avian malaria parasites and other haemosporidia. Boca Raton, FL: CRC Press.

[ece33319-bib-0060] Valkinũas, G. , Zickus, T. , Shapoval, A. P. , & Iezhova, T. A. (2006). Effect of *Haemoproteus belopolskyi* (Haemosporida: Haemoproteidae) on body mass of the blackcap *Sylvia atricapilla* . Journal of Parasitology, 92, 1123–1125. 17152968 10.1645/GE-3564-RN.1

[ece33319-bib-0700] Warner, R. E. (1968). The role of introduced diseases in the extinction of the endemic Hawaiian avifauna. Condor, 70, 101–120.

[ece33319-bib-0061] Wood, M. J. , Cosgrove, C. L. , Wilkin, T. A. , Knowles, S. C. , Day, K. P. , & Sheldon, B. C. (2007). Within‐population variation in prevalence and lineage distribution of avian malaria in blue tits, *Cyanistes caeruleus* . Molecular Ecology, 16, 3263–3273. 17651202 10.1111/j.1365-294X.2007.03362.x

[ece33319-bib-0062] Worms, M. (1972). Circadian and seasonal rhythms in blood parasites In Canning E., & Wright C. (Eds.), Behavioural aspects of parasite transmission (pp. 53–67). London: Academic Press.

[ece33319-bib-0063] Zamora‐Vilchis, I. , Williams, S. E. , & Johnson, C. N. (2012). Environmental temperature affects prevalence of blood parasites of birds on an elevation gradient: Implications for disease in a warming climate. PLoS One, 7, e39208–e39210. 22723966 10.1371/journal.pone.0039208 PMC3378574

