# Supplementary material for: Seasonal dynamics in mosquito abundance and temperature do not influence avian malaria prevalence in the Himalayan foothills
Source: Ecol Evol. 2017 Sep 3;7(19):8040–57. doi: 10.1002/ece3.3319 (PMC5632643; doi:10.1002/ece3.3319)

**Supplementary material:**

**Seasonal dynamics in mosquito abundance and temperature do not influence avian *Plasmodium* prevalence in the Himalayan foothills**

**Farah Ishtiaq^1, 3*^, Christopher G. R. Bowden^2^ and Yadvendradev V. Jhala^3^**

**^1^ Centre for Ecological Sciences, Indian Institute of Science, Bangalore 560012, India**

**^2^Royal Society for the Protection of Birds, The Lodge, Sandy, SG19 3DL, Bedfordshire, UK**

**^3^Wildlife Institute of India, PO Box 18, Chandrabani, Dehradun 248001, Uttarakhand, India**

***Corresponding author: Email:** [**ishtiaq.farah@gmail.com**](mailto:ishtiaq.farah@gmail.com)

Tel: +91 80 22932507

Fax: +91 80 23601428

**Table S1.** Summary of parasite lineages including Genbank accession numbers.

| **Lineage** | **Parasite genus** | **Host Species** | | **Host Family** | **sites** | **GenBank**  **accession no.** |
| --- | --- | --- | --- | --- | --- | --- |
|  |  | **Scientific name** | **Common name** |  |  |  |
| DELURB5 | *Plasmodium* | *Prinia socialis* (2) | Ashy Prinia | Cisticolidae | DUN |  |
|  | *Plasmodium* | *Chrysomme sinense* | Yellow-eyed Babbler | Timaliidae | DUN |  |
|  | *Plasmodium* | *Zosterops palpebrosus* | White-eye | Zosteropidae | DUN |  |
| APR15 | *Plasmodium* | *Prinia socialis* | Ashy Prinia | Cisticolidae | DUN | MF565807 |
| GRW04 | *Plasmodium* | *Prinia socialis* | Ashy Prinia | Cisticolidae | DUN | MF565808 |
|  | *Plasmodium* | *Chrysomme sinense* | Yellow-eyed Babbler | Timaliidae | DUN |  |
| UPUPA01 | *Plasmodium* | *Prinia hodgsoni* | Plain Prinia | Cisticolidae | DUN | MF565809 |
|  | *Plasmodium* | *Orthotomus sutorius* | Tailor bird | Cisticolidae | DUN |  |
| GRW06 | *Plasmodium* | *Stachyris pyrrhops* | Black-chinned babbler | Timaliidae | DUN | MF565810 |
| POMERY01 | *Plasmodium* | *Pomatorhynius erythrogenys* (2) | Rusty-cheeked Scimitar babbler | Timaliidae | DUN | MF565811 |
| PYCAFF01 | *Plasmodium* | *Pycnonotus cafer* (2) | Red-vented Bulbul | Pycnonotidae | DUN | MF565812 |
|  | *Plasmodium* | *Pycnonotus leucogenys* | Himalayan Bulbul | Pycnonotidae | DUN |  |
|  | *Plasmodium* | *Lonchura punctulata* | Spotted Munia | Estrildidae | DUN |  |
| AFTRU5 | *Plasmodium* | *Turdus unicolor* | Tickell’s Thrush | Turdidae | DUN | MF565813 |
| NILSUN01 | *Plasmodium* | *Garrulax albogularis* | White-throated Laughingthrush | Leiothrichidae | CHAK | MF565814 |
| TROLIN02 | *Plasmodium* | *Trochalapteron lineatum* | Streaked Laughingthrush | Leiothrichidae | SHOK | MF565815 |
| TURSTR04 | *Plasmodium* | *Turdoides* *striata* (3) | Jungle Babbler | Timalidae | DUN | MF565819 |
| GW8 | *Plasmodium* | *Phylloscopus trochiloides* | Greenish Warbler | Phylloscopidae | MAG | MF565816 |
| TURSTR02 | *Haemoproteus* | *Turdoides* *striata* (3) | Jungle Babbler | Timalidae | DUN | MF565817 |
| TURSTR03 | *Haemoproteus* | *Turdoides* *striata* | Jungle Babbler | Timalidae | DUN | MF565818 |
| ZOSPAL01 | *Haemoproteus* | *Pomatorhynius erythrogenys* | Rusty-cheeked Scimitar babbler | Timaliidae | DUN | MF565820 |
|  | *Haemoproteus* | *Zosterops palpebrosus* | White-eye | Zosteropidae | DUN |  |
|  | *Haemoproteus* | *Pycnonotus cafer* | Red-vented Bulbul | Pycnonotidae | DUN |  |
| ZOSPAL02 | *Haemoproteus* | *Zosterops palpebrosus*(12) | White-eye | Zosteropidae | DUN | MF565821 |
| ZOSPAL03 | *Haemoproteus* | *Zosterops palpebrosus* | White-eye | Zosteropidae | DUN | MF565822 |
| ZOSPAL04 | *Haemoproteus* | *Zosterops palpebrosus* | White-eye | Zosteropidae | DUN | MF565823 |
| ZOSPAL05 | *Haemoproteus* | *Zosterops palpebrosus*(2) | White-eye | Zosteropidae | DUN | MF565824 |
| PYCAFF02 | *Haemoproteus* | *Pycnonotus cafer* | Red-vented Bulbul | Pycnonotidae | DUN | MF565825 |
| LONPUN01 | *Haemoproteus* | *Lonchura punctulata* (4) | Spotted Munia | Estrildidae | DUN | MF565826 |
| LONPUN02 | *Haemoproteus* | *Lonchura punctulata* | Spotted Munia | Estrildidae | DUN | MF565827 |
| AFR084 | *Haemoproteus* | *Sturnus contra* | Pied Myna | Sturnidae | DUN | MF565828 |
| CENTSIN01 | *Haemoproteus* | *Centropus sinensis* | Coucal | Cuculidae | DUN | MF565829 |
| WTH177 | *Haemoproteus* | *Sylvia curucca* | Lesser Whitethroat | Sylviidae | DUN | MF565830 |
| ACDUM1 | *Haemoproteus* | *Acrocephalus dumetorum* (7) | Blyth’s Reed Warbler | Acrocephalidae | DUN | KY695225 |
|  | *Haemoproteus* | *Carpodacus rodochroa* | Pink-browed Rosefinch | Fringillidae | SHOK |  |
| ACDUM2 | *Haemoproteus* | *Acrocephalus dumetorum* (5) | Blyth’s Reed Warbler | Acrocephalidae | DUN | KY695226 |
| ACDUM3 | *Haemoproteus* | *Acrocephalus dumetorum* (5) | Blyth’s Reed Warbler | Acrocephalidae | DUN | KY695227 |
| MW1 | *Haemoproteus* | *Acrocephalus dumetorum* (2) | Blyth’s Reed Warbler | Acrocephalidae | DUN | KY695230 |
| TROERY01 | *Haemoproteus* | *Trochalapteron erythrocephalum* | Chestnut-crowned Laughingthrush | Leiothrichidae | SHOK | KY623720 |
| GARLAN01 | *Haemoproteus* | *Garrulax lanceolatus* | Black-headed Jay | Timalidae | MAG | MF565831 |
| AEGCON01 | *Haemoproteus* | *Aegithalos concinnus* (2) | Red-headed Tit | Aegithalidae | MAG | MF565832 |
| MYOCA01 | *Haemoproteus* | *Myophonus caeruleus* | Blue Whistling Thrush | Muscicapidae | MAG | MF565833 |
| GW1 | *Haemoproteus* | *Phylloscopus trochiloides* | Greenish Warbler | Phylloscopidae | MAG | MF565834 |
| GW7 | *Haemoproteus* | *Phylloscopus trochiloides* | Greenish Warbler | Phylloscopidae | MAG | MF565835 |
| YWT7 | *Haemoproteus* | *Motacilla cinerea* | Grey Wagtail | Motacillidae | MAG | MF565836 |

* Previously described lineages found are mark in underline. Number of infected individuals per site is indicated in parenthesis. Survey sites are coded: DUN, Dehradun (600m); MAG, Magra (1800m); CHAK, Chakrata (2200m); SHOK, Shokharakh (3200m).

Figure S1 Plot of mosquito abundance (log) and composition by temperature in the foothills of western Himalaya


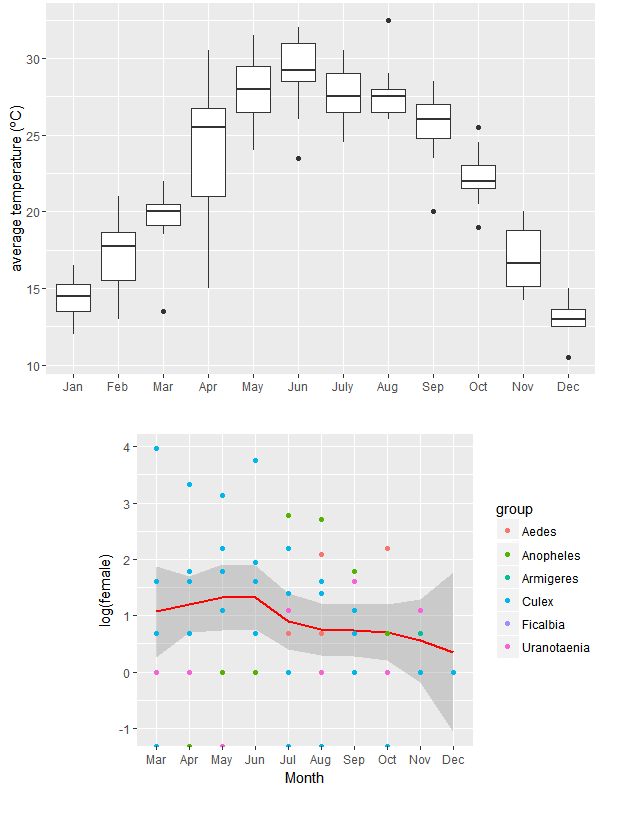

Supplement: Supplementary file 1 [file ECE3-7-8040-s001.doc]
